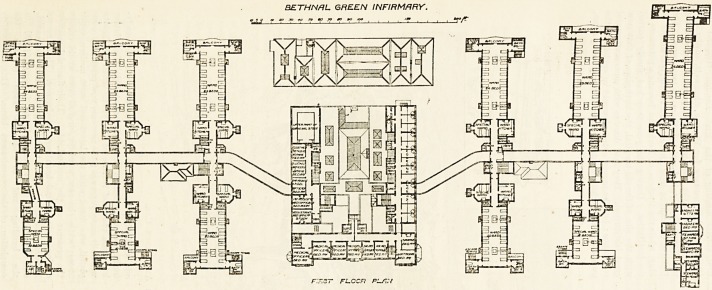# Hospital Construction

**Published:** 1900-03-17

**Authors:** 


### The Institutional Workshop.

### HOSPITAL CONSTRUCTION.

### THE NEW INFIRMARY FOR BETHNAL GREEN.

Several well-designed workhouse infirmaries have lately been erected in the metropolis, and that for Bethnal Green is one of the best of its class which has as yet come under our observation. It is true that the space between the various blocks is scarcely sufficient to find favour in the eyes of the modern hospital expert; but it is equally true that it is no easy matter to get the required number of beds into a building which must be erected on a cramped site in London, where ground space has a fabulous value. Here the site has a length of 740 feet, with a frontage to the Cambridge Road of 330 feet, and to Russia Lane of 280 feet An area of four and a half acres is enclosed, and it must be acknowledged that the architects have made the most of the space at their disposal.

The infirmary consists of fourteen blocks, three of which are for administrative purposes, and eleven for patients' wards. Standing opposite the long axis of the building we should have Cambridge Road on our right hand and Russia Lane on the left, and the administrative block proper would be in front of us. This block is four stories high, and has a basement in addition. It contains rooms for the medical officers, matron, nurses, servants, also the general stores, kitchen, sculleries, &c. Some of these have of necessity to be lighted by open courts. About half way back the main corridor traverses this block, and the kitchen, stores, dairy, &c , are placed in rear of this corridor. There is a strong asylum flavour about this part of the plan. A bridge connects the block with the laundry, and the mortuary and post-mortem are in juxtaposition.

After leaving the administration block the main corridor runs diagonally for about 70 feet, and then resumes its direction parallel with the main frontage. By this method the authors of the plan have obtained almost the same amount of space on each side of the corridor, and the various blocks are placed at right angles to the corridor. These blocks are three stories high, and the floors vary in accommodating from 10 to 36 patients. Excepting in size there is not much difference in the wards. Each is provided with a ward kitchen, a special ward for one bed, a good main staircase, and an extra staircase at the extreme end of the ward. The closets and bath-rooms are in all cases properly cut off by ventilating passages. Bed lifts are provided. Six of the wards have balconies at their south ends, and the main corridor, which is 10 feet wide, has a flat roof permitting its being used as a promenade. One of the blocks in Cambridge Road contains the main entrance, the committee room, clerk's office, &c.

The warming is carried out by central stoves, and these are arranged to introduce warmed fresh air to the wards. Low-pressure hot-water radiators are provided as additional means of warming, but none nor all of these artificial systems can make up for the absence of open fireplaces where sick patients are being treated. All wards are provided with foul-air extracting shafts, and the windows have ventilating hoppers. Tobin's tubes are also used for admitting fresh air. The buildings are of red brick with strings of moulded white Suffolk bricks, and Portland stone is used for the sills and plinths. The corridors, kitchens, and sanitary annexes are faced internally to dado height with glazed bricks. The wards are finished in Keen's cement. Mosaic pavement is used in the corridors, and the ward floors are of pitch pine, wax polished. The total accommodation is for 750 patients, and the cost was £163,000, exclusive of the site. The architects were Messrs. Giles, Gough, and Trollop, and the contractor was Mr. Rowbotham, of Birmingham.

## Figures and Tables

**Figure f1:**
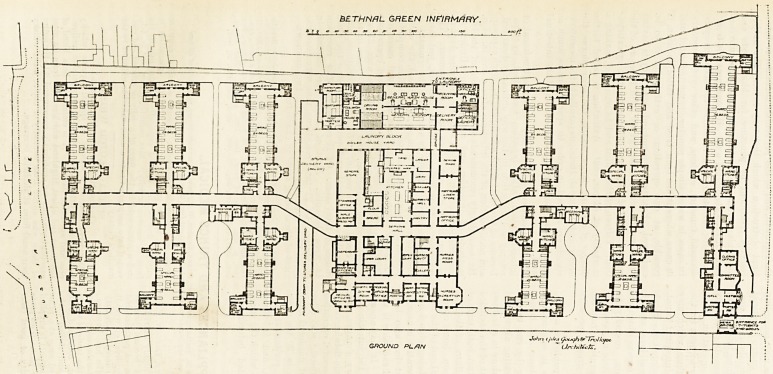


**Figure f2:**